# Coral-Associated Viral Assemblages From the Central Red Sea Align With Host Species and Contribute to Holobiont Genetic Diversity

**DOI:** 10.3389/fmicb.2020.572534

**Published:** 2020-09-30

**Authors:** Anny Cárdenas, Jin Ye, Maren Ziegler, Jérôme P. Payet, Ryan McMinds, Rebecca Vega Thurber, Christian R. Voolstra

**Affiliations:** ^1^Department of Biology, University of Konstanz, Konstanz, Germany; ^2^Red Sea Research Center, Division of Biological and Environmental Science and Engineering (BESE), King Abdullah University of Science and Technology (KAUST), Thuwal, Saudi Arabia; ^3^Department of Animal Ecology & Systematics, Justus Liebig University Giessen, Giessen, Germany; ^4^College of Earth, Ocean, and Atmospheric Sciences, Oregon State University, Corvallis, OR, United States; ^5^Center of Modeling, Simulation and Interactions, Université Côte d’Azur, Nice, France; ^6^Department of Microbiology, Oregon State University, Corvallis, OR, United States

**Keywords:** virus, coral, metagenomics, metatranscriptomics, metaorganism, holobiont, Red Sea

## Abstract

Coral reefs are highly diverse marine ecosystems increasingly threatened on a global scale. The foundation species of reef ecosystems are stony corals that depend on their symbiotic microalgae and bacteria for aspects of their metabolism, immunity, and environmental adaptation. Conversely, the function of viruses in coral biology is less well understood, and we are missing an understanding of the diversity and function of coral viruses, particularly in understudied regions such as the Red Sea. Here we characterized coral-associated viruses using a large metagenomic and metatranscriptomic survey across 101 cnidarian samples from the central Red Sea. While DNA and RNA viral composition was different across coral hosts, biological traits such as coral life history strategy correlated with patterns of viral diversity. Coral holobionts were broadly associated with *Mimiviridae* and *Phycodnaviridae* that presumably infect protists and algal cells, respectively. Further, *Myoviridae and Siphoviridae* presumably target members of the bacterial phyla Actinobacteria, Firmicutes, and Proteobacteria, whereas *Hepadnaviridae* and *Retroviridae* might infect the coral host. Genes involved in bacterial virulence and auxiliary metabolic genes were common among the viral sequences, corroborating a contribution of viruses to the holobiont’s genetic diversity. Our work provides a first insight into Red Sea coral DNA and RNA viral assemblages and reveals that viral diversity is consistent with global coral virome patterns.

## Introduction

Coral reefs comprise one of the most diverse ecosystems in the marine environment. Reef-building corals are the foundation of this ecosystem supporting thousands of animal species (Costanza et al., 1997; Moberg and Folke, 1999; Rohwer et al., 2002). Corals also offer persistent, protected, and nutrient-rich microenvironments to anchor stable partnerships with a wide diversity of microbes. Single-celled dinoflagellates of the family Symbiodiniaceae provide energy for corals to build massive three-dimensional calcium carbonate skeletons (Muscatine et al., 1981; Burriesci et al., 2012), while some bacteria presumably contribute to metabolic cycling and may be involved in immunity and environmental adaptation (Raina et al., 2009; Ziegler et al., 2017; Robbins et al., 2019; Voolstra and Ziegler, 2020). While coral-associated bacterial and Symbiodiniaceae communities have been extensively studied, viral diversity and function have only recently been explored (for an overview consult the following reviews: van Oppen et al., 2009; Vega Thurber and Correa, 2011; Sweet and Bythell, 2017; Vega Thurber et al., 2017).

Early studies in this field used microscopy (Wilson et al., 2004; Davy and Patten, 2007; Lohr et al., 2007), flow cytometry (Seymour et al., 2005; Patten et al., 2006), or amplification- and metagenomics-based genomic analyses (Wegley et al., 2007; Marhaver et al., 2008; Vega Thurber et al., 2008) to show that corals contain a variety of viruses that infect different compartments of the coral holobiont including the associated microalgae, and other organismal entities. Metagenomic surveys of coral viromes have found that single-stranded DNA (ssDNA) viruses are the predominant viral type in coral species from the Great Barrier Reef (Correa et al., 2016; Weynberg et al., 2017a) and the Caribbean Sea (Wegley et al., 2007; Soffer et al., 2014). Phages from the order *Caudovirales* as well as the eukaryotic viral families *Herpesviridae* and *Phycodnaviridae* were shown to consistently associate with corals globally (Wegley et al., 2007; Marhaver et al., 2008; Vega Thurber et al., 2008; Correa et al., 2012; Weynberg et al., 2017a). Further, several studies assessing Symbiodiniaceae cultured isolates found sequences similar to protist and plant infecting viruses in the families *Potyviridae*, *Picornaviridae*, *Herpesviridae*, *Phycodnaviridae*, and *Mimiviridae* (among others) (Brüwer et al., 2017; Lawrence et al., 2017; Weynberg et al., 2017b) and some postulated, a potential role of viruses in the thermotolerance of Symbiodiniaceae (Correa et al., 2012, 2016; Levin et al., 2016; Weynberg et al., 2017b). In addition, metatranscriptomes of the coral model *Exaiptasia pallida* (commonly referred to as *Aiptasia*) were characterized by a high prevalence of the viral families *Herpesviridae*, *Partitiviridae*, and *Picornaviridae*, similar to corals (Brüwer and Voolstra, 2018).

Despite the pervasive abundance of viral particles in marine environments, including coral reefs (Paul et al., 1993; Payet et al., 2014), and their seeming association with corals, even less is known about their functional roles in the coral holobiont. Studies that addressed the function of viruses mostly focus on their detrimental effects (see Vega Thurber et al., 2017 for a review). For instance, herpes-like viruses rapidly increased in response to abiotic stress in corals (Vega Thurber et al., 2008). Further, nucleocytoplasmic large DNA viruses (such as *Phycodnaviridae, Mimiviridae*, and *Iridoviridae*) were found to be more abundant in tissues affected by White Plague Disease compared to the healthy tissue of corals (Soffer et al., 2014). Similarly, bleached corals were found to harbor increased abundances of small circular ssDNA viruses, including *Nanoviridae*, *Circoviridae*, and *Geminiviridae* (Soffer et al., 2014; Correa et al., 2016). This suggests a contribution of viruses to coral bleaching and disease, although it is unclear whether altered viral communities are connected to the underlying cause or simply a consequence of the impacted coral holobiont. Viruses are also proposed to play an important role in environmental acclimatization and adaptation (van Oppen et al., 2009; Levin et al., 2016; Torda et al., 2017). In addition, viruses of coral-associated eukaryotes and bacteria are hypothesized to contribute to holobiont functional diversity. For instance, adherence of some bacteriophages to coral mucosal surfaces regulates the abundance of specific bacteria through targeted infection and lysis, fulfilling an immunity-like function (Barr et al., 2013). Moreover, phage- and virus-induced mortality of bacterial and host cells may contribute to nutrient provisioning within the holobiont (the “revolving door” hypothesis) (Torda et al., 2017; Vega Thurber et al., 2017). Likewise, viral genes encode for auxiliary functions that may be beneficial to the holobiont. For instance, some coral-associated viruses carry genes related to photosynthesis that are suggested to alleviate and/or delay damage to Symbiodiniaceae photosystems from high temperature (Weynberg et al., 2017a).

Regardless of such recent efforts, a basic understanding of viral diversity associated with a phylogenetically diverse set of corals or across regions is unavailable. To date, only a limited number of studies have systematically examined the underlying virome variation among coral species. Thus, to advance knowledge in this field, we here provide the first assessment of viral diversity associated with several coral genera from the central Red Sea. We employed an untargeted metagenomics and metatranscriptomics approach that aimed to avoid known biases and thus more completely describe all viral genome types (RNA- or DNA- based) and viral families associated with a broad coral host range. We generated metagenomes and metatranscriptomes of 101 cnidarian samples collected from 14 different genera in the central Red Sea. Taxonomic assignment of more than 800 million high-quality read pairs (i.e., >1.6 billion sequences, comprising 497 Gb) reveals that coral viromes adhere to coral taxonomical and biological traits.

## Materials and Methods

### Sample Collection, Nucleic Acid Extraction, Sequencing

From twelve scleractinian and two cnidarian outgroups species we collected fragments from five replicate colonies per species via SCUBA on May 17–18, 2016, at Al Fahal reef (N 22.3034, E 38.9602) in the central Red Sea. To best evaluate the diversity of phage and eukaryotic viruses within Red Sea Scleractinia and their relatives, we targeted species that together represent 12 different genera and comprise many coral clades and life history strategies (Table 1). Coral and outgroup specimens were collected using a hammer and chisel or bone cutters and placed in pre-labeled sterile Whirl-Paks. Upon returning to the boat, samples were immediately frozen in a liquid nitrogen dry shipper and then transferred to the laboratory for storage and processing.

**TABLE 1:** Coral species collected from the Red Sea for assessment of associated viruses.

Species	Family (Molecular)	Order	Major clade	Life history strategy	Growth form	Mode of larval development	Sexual system	Substrate	Wave exposure preference	MG	MT
*Acanthastrea echinata*	Lobophyllidae	Scleractinia	robust	Stress-tolerant	massive	spawner	hermaphrodite	attached	broad	X	
*Acropora cytherea*	Acroporidae	Scleractinia	complex	Competitive	tables	spawner	hermaphrodite	attached	broad		X
*Diploastrea heliopora*	Diploastraeidae	Scleractinia	robust	Stress-tolerant	massive	spawner	gonochore	attached	broad	X	X
*Fungia* sp.	Fungiidae	Scleractinia	robust	Stress-tolerant	massive	spawner	gonochore	unattached	broad	X	
*Galaxea fascicularis*	Euphylliidae	Scleractinia	complex	Stress-tolerant	massive	spawner	gonochore	attached	protected	X	X
*Mycedium elephantotus*	Merulinidae	Scleractinia	robust	Generalist	laminar	spawner	hermaphrodite	attached	protected	X	X
*Pachyseris speciosa*	Pachyseridae	Scleractinia	complex	Generalist	laminar	spawner	gonochore	attached	broad	X	X
*Pavona varians*	Agariciidae	Scleractinia	complex	Stress-tolerant	encrusting	spawner	gonochore	attached	broad	X	
*Plerogyra sinuosa*	Plesiastreidae	Scleractinia	robust	Stress-tolerant	massive	spawner	hermaphrodite	attached	protected	X	
*Pocillopora verrucosa*	Pocilloporidae	Scleractinia	robust	Competitive	branching	spawner	hermaphrodite	attached	broad	X	X
*Porites lutea*	Poritidae	Scleractinia	complex	Stress-tolerant	massive	spawner	gonochore	attached	protected	X	X
*Stylophora pistillata*	Pocilloporidae	Scleractinia	robust	Weedy	branching	brooder	hermaphrodite	attached	exposed	X	X
*Millepora platyphylla*	Milleporidae	Anthomedusae	NA	NA	NA	NA	NA	NA	NA	X	
*Xenia* sp.	Xeniidae	Octocorallia	NA	NA	NA	NA	NA	NA	NA	X	

*NA, not applicable. Coral biological traits were taken from the Coral Trait Database (Madin et al., 2016). Traits for Fungia were annotated from Fungia fungites. Reproduction traits for Acropora cytherea and Plerogyra sinuosa were missing from the coral trait database and therefore taken from previous reports (Mundy and Babcock, 2000). Specimens were collected from 12 scleractinian corals from the robust and complex clades and 2 outgroups. Scleractinian corals fall within 11 families and represent four different functional groups.*

In total, 70 field samples were collected, consisting of 5 biological replicates (individual cnidarian colonies) per species × 14 species. Nucleic acids for all 70 field samples were extracted and processed for metagenomes but three metagenomes were excluded due to low amount of DNA extracted, resulting in 67 metagenomics libraries. A subset of 40 coral samples, consisting of 5 biological replicates (individual coral colonies) per species × 8 species were processed for metatranscriptomes, but six samples were excluded due to low amount of RNA extracted, resulting in 34 metatranscriptomic libraries. A total of 101 cnidarian samples (67 metagenomes and 34 metatranscriptomes) were generated from the 70 field samples, and of those, a total of 57 metagenomes plus 29 metatranscriptomes were included in the analysis.

DNA and RNA were extracted from 70 cnidarian fragments using the RNA/DNA AllPrep Kit (Qiagen, United States) with modifications. For DNA isolation, coral fragments were defrosted, and coral surface mucus layer and tissue were blasted off from the skeleton using a pressurized air gun. Then ca. 30 mg of air-blasted tissue slurry was weighted out in a sterile weight boat and 600 μl of lysis buffer (RLT buffer by Qiagen) was added to the slurry. The mixture was homogenized with a sterile 20-gauge needle on a 1-ml syringe and transferred in a sterile 2-ml tube. DNA was then extracted as recommended by the manufacturer. For RNA isolation, frozen samples were placed in pre-cooled and sterile mortars and pulverized in the presence of liquid nitrogen. Thus, RNA samples contain nucleic acids from the coral skeleton, mucus and tissue, while DNA samples contain nucleic acids from the coral mucus and tissue. A 2-ml volume of lysis buffer and beta-mercaptoethanol solution were added to the still frozen slurry, and samples were then homogenized with a 20-gauge needle on a 1-ml syringe. RNA was then extracted as recommended by the manufacturer. All DNA and RNA extractions were quantified on a Qubit (Thermo Fisher Scientific, United States) prior to sequencing library preparation.

Metagenomic libraries were generated using the NEBNext Ultra II DNA Library Kits and 100 ng of DNA per sample. First the DNA was sheared to approximately 250–300 bp (Covaris M2). Then, sheared DNA was end-repaired and A-tailed with a single adenine. Following this, adapters were ligated to the end-repaired A-tailed DNA fragments. For size selection of 250 bp fragment inserts, Agencourt AMPure XP beads (Beckman Coulter) were used. Library enrichment was conducted with Illumina TruSeq HT indexes (dual index) using six cycles of PCR. For quality control of the libraries a Bioanalyzer DNA 1000 Chip (Agilent Technologies) was used, followed by quantification using the Qubit BR DNA system (Invitrogen), and subsequent pooling in equimolar ratios to a final concentration of 10 nM. Lastly, the pooled libraries were re-quantified using qPCR (KAPA Biosystems library quantification on ABI HT7900, Applied Biosystems) and paired-end sequenced (2 × 300 bp) on the Illumina NextSeq 500 platform at 1.8 pM with 1% PhiX.

Metatranscriptomic libraries were generated using the NEBNext Ultra Directional RNA Library kit. About 100 ng of total RNA were fragmented to an approximate final size of 300 bp, equating to an incubation time of 10 min at 94°C. The manufacturer’s protocol was followed for the remainder of the steps. Size selection of the library was performed using AMPure XP beads for size selection of 300 bp fragment inserts. Dual indexes were used in the library enrichment step. The library was enriched using 12 cycles of PCR. Libraries were quality checked using the Agilent Bioanalyzer DNA 1000 Chip. Libraries were quantified using the Invitrogen Qubit BR DNA system and pooled in equimolar ratios to a final concentration of 10 nM. The pool was re-quantified using qPCR, KAPA Biosystems Library quantification, using the ABI HT7900. Libraries were paired-end sequenced (2 × 300 bp) on the Illumina NextSeq500 platform at 1.8 pM with 1% PhiX.

### Data Analysis

Illumina adaptors and low-quality reads (quality score below 15, length below 40 bp) were removed using Trimmomatic v0.36 (Bolger et al., 2014). We first aimed for a contig-based approach using MEGAHIT v1.1.1-2 (Li et al., 2015), but a large fraction of putative viral reads remained unassembled and therefore we opted for a read-based approach using Kaiju (see below). To explore overall taxonomic read distribution we used CCMetagen v1.1.5 (Marcelino et al., 2020). The majority of reads were annotated to Anthozoa (86.46% of annotated sequences in coral samples) and Hydrozoa (38.89% of annotated sequences in outgroup samples), and to a lower extend Dinophyceae (6.18% of annotated sequences across all samples) (Supplementary Figure S1A and Supplementary Table S5). Thus, as expected, a majority of sequence reads stem from the respective cnidarian host or the associated microalgae. To prepare data for analysis in Kaiju, we removed cnidarian read pairs using bbsplit function from BBMap v38.24 (Bushnell, 2014) against a collection of sequences and genomes belonging to the NCBI taxonomy ID 6073 (Cnidaria) deposited in the database. Metatranscriptomic read pairs were additionally compared against the SILVA reference database release 132 (Quast et al., 2013) using SortMeRNA v2.1b (Kopylova et al., 2012) to remove rRNAs. The remaining metagenomic and metatranscriptomic read pairs were taxonomically annotated using the maximum exact matches (MEMs) mode of Kaiju v1.7.2 with a minimum match length of 11 (Menzel et al., 2016). We queried the NCBI BLAST nr_euk database that includes all proteins belonging to viruses, archaea, bacteria, dinoflagellates, and other microbial eukaryotes (2019-06-25).

Metagenomes and metatranscriptomes with no replicates or with less than 50 read pairs annotated as viral were removed from downstream analyses (underlined samples shown in Supplementary Tables S1, S2) as well as taxa with relative abundance lower than 0.1%. Viral family abundances were normalized by sequencing depth, and statistical analyses and plotting were performed with R v3.4.2 (R Development Core Team, 2010). Average relative abundances of sequences annotated to the most abundant 20 viral families for each coral species were represented as bar plots using ggplot2 v3.0.0 (Wickham, 2016). Observed number of families were calculated using the estimateR function of the package Vegan (Oksanen et al., 2013). Shapiro–Wilk tests confirmed alpha diversity estimate values were not normally distributed. Overall significant differences were calculated with Kruskal–Wallis tests and pairwise *post hoc* comparisons were done using the Dunn’s test. *P*-values from multiple testing were adjusted using the False Discovery Rate (FDR) correction for multiple testing. The variation on viral communities was evaluated by pairwise Permutational Multivariate Analysis of Variance (PERMANOVA) using the wrapper function “pairwise.adonis” (Arbizu, 2019) for multilevel pairwise comparison from “adonis” function of the package Vegan in R. Hierarchical clustering was done using Ward’s minimum variance method using the “hclust” function. A distance-based redundancy analysis (RDA) was performed using Bray-Curtis dissimilarity matrices of log10(1 + x) transformed viral family abundances using “capscale” from Vegan implemented in Phyloseq (McMurdie and Holmes, 2013). RDAs of log10(1 + x) transformed viral family abundances were independently constrained to taxonomical and biological host traits and therefore the sum of their inertia values lead to >100%. We compared the relative influence of each trait on viral diversity by z-score normalizing inertia scores within traits and representing them as heatmaps for the purpose of depicting which traits have stronger influence on viral assemblages. Biological traits associated with each coral species were annotated from the Coral Trait Database (Madin et al., 2016). To represent the contribution of viral families between coral host species and life history strategies, a principal component analysis (PCA) biplot was done. Species scores were indicated by biplot arrows, obtained from unconstrained distance-based RDAs using the package Vegan. Log10(x + 1)-transformed abundances of microbial and the most abundant 20 viral families were used to calculate the non-parametric Spearman’s rank correlation coefficient using the “rcorr” function of the Hmisc package (Harrell and Harrell, 2019). Reads pairs that were classified as virus by Kaiju were merged and functionally annotated against the SEED subsystems database (Overbeek et al., 2005) using the Metagenomics RAST server (MG-RAST) (Meyer et al., 2008). Additionally, viral sequences were annotated against viral protein databases from UniProt (Swiss Prot: 17,008 protein sequences and TrEMBL: 4,480,041 protein sequences as of Aug 5th, 2020) using a translated nucleotide query. The search was done using MMseqs v11-e1a1c (Steinegger and Söding, 2017) only allowing hits with *e*-values < 0.001 and bitscores >30. UniProt annotations were mapped to Gene Ontology (GO) terms that were summarized in semantic similarity-based tree-maps in REVIGO (last update Jan 2017) (Supek et al., 2011) and visualized using CirGO v2.0 (Kuznetsova et al., 2019).

## Results

### Viral Sequencing Overview

In this study we characterized taxonomic and functional viral consortia from a diverse set of Red Sea corals using metagenomic and metatranscriptomic sequencing of whole-tissue preparations. A total number of 828 million read pairs (i.e., ∼1.6 billion paired-end reads) were obtained across 67 metagenomic and 34 metatranscriptomic samples, comprising 14 cnidarian species (12 corals and two outgroups, see Table 1 and Supplementary Tables S1, S2). A total of 780 million read pairs passed quality control (Supplementary Tables S1, S2). After removing cnidarian sequences from all samples and rRNA sequences from metatranscriptomic samples, on average ∼232 million and ∼1.3 million read pairs were retained per sample for metagenomes and metatranscriptomes, respectively. A total of 49,998 (0.01%) and 16,169 (0.004%) viral read pairs were identified in 57 metagenomes and 29 metatranscriptomes, respectively. This corresponds to an average of 877 and 558 viral read pairs per sample in metagenomes and metatranscriptomes, respectively, that were used for subsequent analyses.

### Viral Community Composition of Red Sea Corals

**FIGURE 1:**
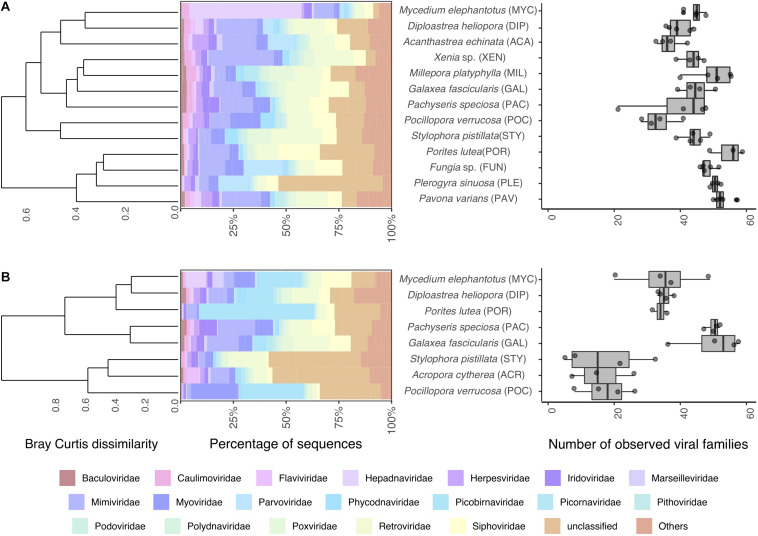
Coral viral community composition and diversity. Taxonomic profile of viral reads classified to the family level **(left)** and estimated number of viral families **(right)** found in coral **(A)** metagenomes and **(B)** metatranscriptomes. Barplots represent mean abundances of the 20 most abundant viral families annotated. Dendrograms represent hierarchical clustering on Bray-Curtis dissimilarities using Ward’s minimum variance method. Boxplots denote observed viral families.

To gain further insight into the viral diversity of Red Sea corals, we first assessed relative abundances of the 20 most abundant viral families in addition to all other families aggregated under the common category “others” (Figure 1A). A total of 117 viral families were found across all samples, 95 in metagenomes and 114 in metatranscriptomes, and 92 were shared between metagenomes and metatranscriptomes. The median number of families found per sample was 46 in metagenomic samples and 34 in metatranscriptomes (Figure 1B). Overall viral diversity was significantly different between metagenomes and metatranscriptomes across all samples (PERMANOVA, R2 = 0.07, *P* < 0.05, Supplementary Figure S3) and pairwise comparisons revealed differences between viral composition in metagenomes and metatranscriptomes of *Diploastrea heliopora, Mycedium elephantotus, Pocillopora verrucosa*, and *Stylophora pistillata* (Supplementary Table S3). Significant higher viral family richness was observed in metagenomes compared to metatranscriptomes in *Porites lutea*, and *S. pistillata* (pairwise *t*-tests, all *P* < 0.01, Supplementary Table S4). The most abundant viral families were *Mimiviridae, Retroviridae*, and *Siphoviridae*, that represent mostly dsDNA viruses infecting eukaryotes and bacteria (Supplementary Figure S2). Notably, many of the most abundant viral families were shared between metagenomes and metatranscriptomes. However, some of the low-abundance viral families were only present in either metagenomes or metatranscriptomes, partially due to the differences in the nucleic acid composition (RNA or DNA) of the putative viral genome (Figure 1). For instance, *Qinviridae, Nyamiviridae*, and *Solinviviridae*, all negative-sense ssRNA viruses, were only present in metatranscriptomes, while 22 viral families were only present in metagenomes. Notably, in the metagenomic dataset, the cnidarian outgroups *Millepora platyphylla* and *Xenia* sp. had distinct viral communities from each other (PERMANOVA test: *F* = 2.83, *P* = 0.01), and only *M. platyphylla* had a different viral composition in comparison to scleractinian corals (PERMANOVA test, *F* = 4.31 *P* = 0.01).

### Drivers of Coral-Associated Viral Diversity

**FIGURE 2:**
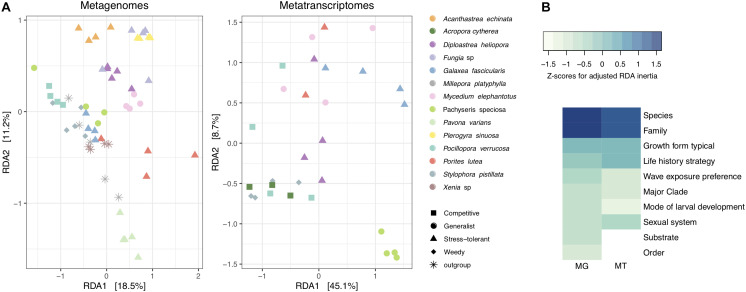
Alignment of coral viral community composition and host traits. **(A)** Log10(1 + x)-transformed viral abundances of coral metagenomes and metatranscriptomes were constrained by host species using a distance-based redundancy analysis (RDA). Colors represent coral species and shapes represent host life history strategies. The percent variation explained by the RDA ordinations is indicated on the axes. **(B)** Heatmap of RDA inertia values for independent comparisons on each coral trait and the proportion of variance explained with regard to viral composition. Inertia values are Z-scaled and normalized within rows. MG stands for metagenomes and MT stands for metatranscriptomes.

To determine which coral traits were the most significant to explain differences in viral composition, a series of constrained RDA analyses were performed (Supplementary Table S5). We compared the relative influence of coral traits by Z-score normalizing RDA inertia values (i.e., variation explained) across factors to elucidate which traits explain most of the variation in viral assemblages between coral hosts. Coral taxonomic traits, particularly at lower ranks such as species and family, explained a large fraction of the variation of viral communities in metagenomes and metatranscriptomes (Figure 2). Host species identity explained the highest proportion of the variance in metagenomes (RDA, constrained inertia = 62.44%) and metatranscriptomes (RDA, constrained inertia = 64.51%) (Figure 2 and Supplementary Table S5). Among coral biological traits, “growth form” and “life history strategy” explained the highest proportion of the variance in metagenomes (RDA, constrained inertia = 28.00%) and metatranscriptomes (RDA, constrained inertia = 37.96%). Notably, however, besides “growth form” and “life history strategy,” the remaining coral biological traits explained only little of the variation in viral composition (Figure 2 and Supplementary Table S5).

To elucidate which viral families contributed to differences in viral community between coral species, growth form, and life history strategies, we projected viral family scores obtained by RDA on PCA biplots (Supplementary Figure S4). For the metagenomes, the viral families *Hepadnaviridae, Parvoviridae*, and *Mimiviridae* had the highest scores (RDA family scores = 175.74, 107.14, and 97.94, respectively) to explain differences between coral species, life history strategies, and growth forms. For instance, the viral family *Parvoviridae* was more abundant (relative abundances from 5 to 16%) in viromes of the stress-tolerant corals with massive growth (*A*ca*nthastrea echinata*, *D. heliopora, Fungia* sp., and *Plerogyra sinuosa)* in comparison to those with other life history strategies and growth forms (relative abundances from 0 to 2%). Similarly, higher relative abundances of the viral family *Dicistroviridae* were observed in the outgroups *M. platyphylla* and *Xenia* sp. as well as in *S. pistillata*, representing the weedy life history strategy (relative abundances between 1 and 3%) compared to the rest of the coral viromes (relative abundances <1%). In metatranscriptomic samples *Picobirnaviridae* and *Siphoviridae* (RDA family scores = 0.97 and 0.79, respectively) accounted for most of the variation between viromes of *Galaxea fascicularis, M. elephantotus*, and *Pachyseris speciosa* (9% average relative abundance) in comparison to *Acropora cytherea*, *P. verrucosa*, and *S. pistillata* (1.4% average relative abundance), resulting in differences between competitive and weedy strategist coral species with branching and table growth versus generalists and stress-tolerant corals with laminar and massive growth (Supplementary Figure S4).

To identify potential viral-host associations of members of the coral holobiont, we calculated the non-parametric Spearman’s rank correlations between the abundance of bacterial and microbial eukaryotic hosts and viral families (Supplementary Figures S1B–D and Supplementary Table S6). Phages from the family *Siphoviridae* showed strong positive correlations (Spearman’s *R* > 0.8, *P*-value < 0.01) with bacterial families (*n* = 37), mainly affiliated to the phyla Firmicutes and Actinobacteria. Similarly, phages of the *Podoviridae* family showed strong correlations with 10 bacterial families, mostly members of the Proteobacteria and Actinobacteria phyla (Supplementary Table S6). In addition, viruses infecting invertebrates and unicellular eukaryotes showed strong positive correlations (Spearman’s *R* > 0.8, *P*-value < 0.01) with 429 families of microbial eukaryotes in the dataset. Members of the *Mimiviridae, Baculoviridae, Pithoviridae, Flaviviridae*, and *Iridoviridae* showed strong positive correlations with members of the class Agaricomycetes (Kingdom: Fungi, Phylum: Basidiomycota) followed by members of the classes Sordariomycetes (Kingdom: Fungi, Phylum: Ascomycota) and Mucoromycetes (Kingdom: Fungi, Phylum: Mucoromycota) (Supplementary Table S6).

### Viral Functional Diversity of Cnidarian Metagenomes and Metatranscriptomes

**FIGURE 3:**
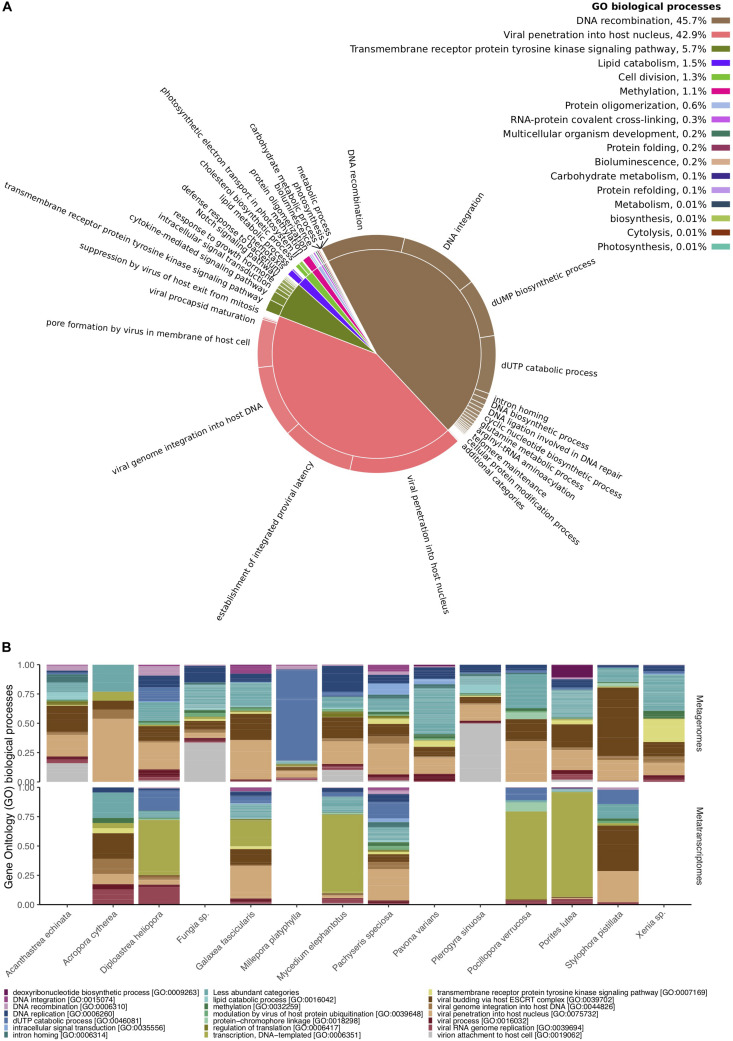
Functional diversity of Red Sea coral-associated viruses. **(A)** Gene Ontology (GO) enriched biological processes in a two-level hierarchical structure. Contribution of higher order processes provided in percent at the top right; relative contribution of lower order provided in form of a pie chart. **(B)** Relative abundances of the 20 most abundant GO biological processes annotated to viral sequences across cnidarian metagenomes **(top)** and metatranscriptomes **(bottom)**.

From the 49,998 metagenomic and 16,169 metatranscriptomic viral paired reads, 1,451 and 343 merged sequences showed similarities to the SEED database, respectively, and 18,260 and 6,068 to UniProt databases, respectively (Supplementary Tables S1, S2). The two most common functional categories annotated to the SEED database were “Virulence, Disease, and Defense” and “Phages, Prophages, Transposable elements, and Plasmids” (Supplementary Table S7). Bacteriophage structural proteins accounted for 37% of the annotated viral sequences in metagenomes and 61% in metatranscriptomes, whereas genes related to *Streptococcus* virulomes accounted for 40% of the annotated viral sequences in metagenomes and only 9% in metatranscriptomes (Supplementary Table S7). Genes related to nucleoside and protein metabolism, motility and chemotaxis, photosynthesis, amino acids and vitamin synthesis, were less frequent and accounted for an additional 20% in metagenomes and 30% in metatranscriptomes (Supplementary Table S7). Similar patterns were found in UniProt annotations (Supplementary Tables S8, S9 and Figure 3). Viral proteins involved in DNA integration and recombination, and those involved in viral penetration into host cells, comprised the most common biological processes found across all samples (Figure 3A). Notably, metagenomes were enriched for viral proteins involved in DNA integration, recombination, and replication, while most metatranscriptomes contained a large proportion of proteins involved in DNA transcription and viral RNA replication (Figure 3B). Sequences encoding for proteins involved in metabolic processes (e.g., carbohydrate metabolism), photosynthesis, bioluminescence and chemotaxis were found at much smaller frequencies (less than 0.01%) (Supplementary Table S9 and Figure 3A).

## Discussion

Despite an increase of studies investigating viral diversity associated with corals [reviewed in Wood-Charlson et al. (2015); Sweet and Bythell (2017), Vega Thurber et al. (2017)], the identification of viral sequences in metagenomic and metatranscriptomic data is still challenging due to the limited amount of viral sequence reference data available (Edwards and Rohwer, 2005; Brüwer et al., 2017; Brüwer and Voolstra, 2018). At the time of analysis (19 March 2020), 63,337,142 sequences were deposited in the NCBI nucleotide database for bacteria while only 3,295,539 sequences for viruses (∼5% compared to bacteria) were available. Besides the paucity of reference databases for virus annotation, uncertainties associated with a lack of homology at the nucleotide level need to be considered.

In this study we present the first exploratory insight of Red Sea coral viruses across a diverse range of coral species. A caveat to our analysis is the low viral coverage compared to coral, microalgae, and bacteria, but we made a conscious decision to trade in sequencing coverage for reducing known bias stemming from enrichment methods (Yilmaz et al., 2010; Kim and Bae, 2011; Wood-Charlson et al., 2015). Our approach provided the possibility to analyze viral diversity independently from known bias(es) caused by fractionation, and further, the possibility to integrate meta-omics data from the same sample (e.g., for correlative analyses to propose viral-host pairings). Furthermore, while the assembly of metagenomic reads into contigs offers many advantages for taxonomic and functional annotation, the effectiveness of this approach depends on the sequencing coverage and complexity of the sample. Coral metagenomes and metatranscriptomes are challenging due to pervasive coral host representation and high and heterogenous sequence diversity that only favors the assembly of the most abundant representatives from the total community, while at the same time it limits the resolution of the compartments with lower biomass such as bacteria and viruses. On the other hand, read-based approaches are proven to be effective to create accurate taxonomical and functional profiles from complex environments where it is not always feasible to produce high quality metagenomes or metatranscriptomes (Beinart et al., 2018; Liu et al., 2019). Here we used the maximum exact match at the protein level as implemented in Kaiju for annotation (Menzel et al., 2016). This method can increase annotation sensitivity by a factor of 10 in comparison to k-mer based nucleotide matching methods (Menzel et al., 2016). From the initial ∼800 million read pairs, Kaiju annotated 50,122 read pairs in metagenomes and 16,254 read pairs in metatranscriptomes as viruses across all 86 cnidarian samples. Thus, given the high number of coral species and their viral sequence representation assessed here, this comprises the most replicated coral virome dataset to date.

### Red Sea Corals Are Dominated by Transcriptionally Active dsDNA Viruses

The viral families *Mimiviridae, Myoviridae, Retroviridae*, and *Siphoviridae* were ubiquitous and abundant in metagenomes and metatranscriptomes. Notably, these families are not only abundant in Red Sea corals, but they are also associated with corals from diverse locations such as the Great Barrier Reef (Weynberg et al., 2017a), Hawaii (Vega Thurber et al., 2008), Florida (Correa et al., 2012), and the Eastern Caribbean (Soffer et al., 2014). This argues for a common set of viral families associated with corals globally.

Among the most abundant viral families observed in Red Sea corals, members of the *Mimiviridae* are known to infect algae and putatively infect coral endosymbionts of the family Symbiodiniaceae (Correa et al., 2012; Weynberg et al., 2017b). Further, members of the family *Phycodnaviridae* (Correa et al., 2012, 2016; Levin et al., 2016) are suggested to be implicated in coral bleaching (Marhaver et al., 2008; Correa et al., 2016). Families from the largest order of phages, the Caudovirales (e.g., *Siphoviridae, Podoviridae*, and *Myoviridae*), are among the most abundant viruses identified in the Red Sea corals assessed here, and constitute a dominant viral group in most coral species (Soffer et al., 2014; Weynberg et al., 2017a) as well as in other marine invertebrates, such as sponges (Laffy et al., 2016, 2018). Caudovirales are comprised of tailed bacteriophages that infect a wide range of bacterial hosts, including members of the *Pseudoalteromonas* (Wichels et al., 2002), *Vibrio* (Kim et al., 2012; Sun et al., 2019), and Rhodobacteraceae (Huang et al., 2011), which are all common coral-associated bacterial taxa. Some members of the Caudovirales are prevalent in bleached corals (Marhaver et al., 2008; Littman et al., 2011; Correa et al., 2016), suggesting that phage infections contribute to the physiology of the coral holobiont.

We also found a large proportion of viruses (*Retroviridae, Hepadnaviridae Parvoviridae, Iridoviridae*, and *Herpesviridae* among others) known to target invertebrate and vertebrate hosts (∼50% of the most abundant 20 families), which we presume to target the coral host. Although some of these are generally considered vertebrate viruses, exceptions have been identified in various invertebrates (Leblanc et al., 1997; Davison et al., 2005; Gudenkauf et al., 2014), including corals and even Symbiodiniaceae cultures (Weynberg et al., 2017b), where putative vertebrate-specific viruses were found to be widely associated. These viral families are often linked with coral stress. For instance, herpes-like viruses increase their abundance during heat and nutrient stress (Vega Thurber et al., 2008; Correa et al., 2016), members of the *Circoviridae* are associated with White Plague Disease in Caribbean corals (Soffer et al., 2014), and *Iridoviridae*-like viruses were found associated with White Syndrome (Patten et al., 2008). In our study, *Iridoviridae* and *Herpesviridae* were among the 10 most abundant families across metagenomes and metatranscriptomes, indicative of their cosmopolitan prevalence and transcriptional activity in seemingly healthy Red Sea corals. This is consistent with the hypothesis presented by Soffer et al. (2014) that herpes-like viruses are implicated in long-term and non-fatal infections in corals, similarly to their infections of vertebrate hosts.

Analysis of metagenomic and metatranscriptomic viromes evidenced that dsDNA viruses were prevalent in both types of samples, most likely, as a consequence of the high transcriptional activity of DNA viruses. Apart from this, differences in viral diversity of metagenomes and metatranscriptomes were observed (Supplementary Figure S3), suggesting absence of a strong methodological bias previously observed in pre-fractionated or chloroformed samples (e.g., predominance of ssDNA viruses) (Wegley et al., 2007; Marhaver et al., 2008). In addition, most studies report a lower occurrence of RNA viruses in most marine invertebrate systems (Shi et al., 2016), which might be attributed to the circumstance that the majority of studies focus on metagenomes for the study of viral communities. However, we cannot rule out that limited representation of RNA viruses in the database queried (NCBI nr + microbial eukaryotes, see above) contributes to this observation. In any case, viral diversity analyses of metagenomes and metatranscriptomes is informative and complementary allowing for direct links between presence and activity of certain viruses. For instance, members of the *Myoviridae* (dsDNA) were found in higher relative abundances in metatranscriptomes compared to metagenomes of *P. verrucosa* suggesting their high transcriptional activity. Similarly, members of the *Hepadnaviridae* (partially dsDNA) were dominant in metagenomic data of *M. elephantotus*, but their low abundance in metatranscriptomes suggests that they are not particularly active. Notably, due to their reverse transcription activity during replication (Nassal, 2008), *Hepadnaviridae* RNA intermediates are converted into cDNA, causing a potential underestimation of this family in metatranscriptomes.

Despite the high prevalence of DNA viruses in metatranscriptomes, we could evidence a predominance of some RNA viruses, such as the dsRNA viral family *Picobirnaviridae* in *D. heliopora*, *M. elephantotus, P. verrucosa*, and *P. lutea*. Members of this viral family are shown to be associated with invertebrates (Shi et al., 2016) therefore, they presumably infect corals.

### Coral Species Harbor Different Viral Assemblages That Reflect Coral Biology and Microbial Diversity Patterns

We found that patterns of viral diversity correlated best with coral host species or family, and conversely, very little with higher taxonomic categories (i.e., coral clade or order). Besides the general notion of coral host-specificity that is supported by this observation, we accounted for several cases where distant coral species harbored similar viral communities. In these cases, the similarity in associated viruses may arise from shared biological traits. For instance, our results suggest that competitive and weedy corals have similar viral assemblages that are distinct from stress-tolerant and generalist corals in metatranscriptomes and metagenomes, even though the coral species considered span large taxonomic distances. In the same way, corals with laminar and massive growth forms have similar viral diversity that differs from corals with branching and laminar growth forms. Given that growth form is an important trait for defining coral life history strategy, our results suggest that growth form is the individual biological trait that best correlates with viral diversity.

On the other hand, biotic factors are key to understand viral diversity as viral communities drive and are driven by the diversity of their hosts. To provide insight on the role of viruses to influence microbial communities, we used Spearman’s correlative associations to infer potential viral-host pairings in the coral holobiont. Our results show that phages from the families *Siphoviridae* and *Podoviridae* possibly infect members of the bacterial phyla Actinobacteria, Firmicutes, and Proteobacteria and have a broader range of bacterial hosts in comparison to other viral families of the order Caudovirales, suggesting better adaptability to exploit novel hosts within the coral holobiont. Most viral-eukaryote pairings involve the viral families *Mimiviridae* and *Baculoviridae* with several families of the classes Agaricomycetes and Sordariomycetes, previously reported as dominant fungal taxa in corals (Amend et al., 2012). *Mimiviridae* and *Baculoviridae* are known to infect unicellular eukaryotes and members of the phylum Arthropoda, respectively. Notably, the full range of hosts infected by *Mimiviridae* is probably underestimated, given that studies have suggested corals and sponges as *Mimiviridae* hosts (Claverie et al., 2009). While we cannot discard the possibility that unicellular fungi are *Mimiviridae* hosts, another explanation for our results is that these fungal associates live in a close relationship with coral-associated Arthropoda and therefore positively correlated with Arthropoda viruses. In addition, the absence of correlations between viral abundances and the algal endosymbionts (Symbiodiniaceae) or the pervasive bacterial taxa *Endozoicomonas* (Endozoicomonadaceae), suggests complex viral-host interactions that likely involve non-linear changes in abundances of more than one viral family.

### Red Sea Coral-Associated Viruses Encode for Virulence-Related, Metabolism, and Photosynthesis Proteins

Viruses can provide a plethora of benefits to their eukaryotic and prokaryotic hosts, e.g., in the form of lysis-derived nutrient cycling, modulation of host gene expression, or horizontal gene transfer (Rohwer and Vega Thurber, 2009; Roossinck, 2011; Obeng et al., 2016). In particular, viral-mediated gene transfer can profoundly impact the way in which host cells interact with their environments (Ochman et al., 2000; Brüssow et al., 2004) in addition to expanding the genetic diversity of the holobiont (Weynberg et al., 2017a; Laffy et al., 2018). For instance, horizontally aquired genes encode for toxins, antibiotic resistance, or photosystem genes (among others) and can provide a (temporal) selective advantage through supporting the colonization of tissues or supplementing the host’s photosynthesis (Ochman et al., 2000; Brüssow et al., 2004; Lindell et al., 2005, 2007; Sullivan et al., 2006).

Our results show that genes involved in metabolism, bacterial motility, and photosynthesis were consistently found in Red Sea coral metagenomes and metatranscriptomes. Viral genes annotated to photosynthesis encode for homologs of the photosystem II, *psbA* (protein D1), and *psbD* (protein D2), often found in phages, especially cyanophages (Sullivan et al., 2005, 2006; Ruiz-Perez et al., 2019). Evidence for the presence of *psbA* and *psbB* have been found in coral viromes from the Caribbean (Marhaver et al., 2008) and the Great Barrier Reef (Weynberg et al., 2017a) as well as from metatranscriptomes of corals infected with Black Band Disease (Garcia et al., 2016). Viral-mediated transduction of photosynthetic genes has been suggested to alleviate the damage to photosystem II of Symbiodiniaceae and photosynthetic bacteria during heat stress (Weynberg et al., 2017a). The high diversity of auxiliary processes linked with viral sequences suggests that viral assemblages are important contributors of genetic diversity to coral holobionts. Genes related to bacterial virulence were the most common within Red Sea coral viromes, suggesting that viruses engage in active genomic exchange with bacteria. Transfer of genes related to virulence plays an important role in the emergence of pathogenic bacterial strains (Examples discussed in Ochman et al., 2000; Brüssow et al., 2004). In fact, some *Vibrio* species have acquired their virulence genes from phages (Jermyn and Boyd, 2002; Khemayan et al., 2012). Coral-derived viral sequences obtained in this study had the highest similarity to “*Streptococcus pyogenes* virulomes.” Such gene families are conserved among strains and characterized by the presence of a wide array of exotoxins, adhesins and invasins, proteases, and many other genes (Ibrahim et al., 2016). Phage-mediated transduction has been previously suggested to be a main driver of coral-associated bacterial virulence. For instance, transcriptomes of the bacterial compartment of the coral *Orbicella faveolata* during White Plague Disease were enriched for phage transcription factors and staphylococcal associated pathogenicity island (SaPI) genes (Daniels et al., 2015). In addition, Weynberg et al. (2015) proposed that phages mediate the transfer of homologs of virulence genes of the human pathogen *Vibrio cholerae* to some strains of the coral-associated *Vibrio coralliilyticus*, by demonstrating that phages encode homologs of those virulence genes. In the same way, a viral origin was suggested for several virulence genes found in metatranscriptomes of coral reef bacterioplankton populations (Cárdenas et al., 2017).

## Data Availability Statement

Sequence data determined in this study are accessible at NCBI under BioProject ID PRJNA437202 (http://www.ncbi.nlm.nih.gov/bioproject/437202) for the metagenomes and BioProject ID PRJNA638633 (https://www.ncbi.nlm.nih. gov/bioproject/PRJNA638633) for the metatranscriptomes. Scripts used for data analysis are available at: https://github.com/ajcardenasb/Red_Sea_virome.

## Author Contributions

JP, RM, RVT, and CRV conceived and designed the experiments. MZ, JP, RM, and RVT generated the data. AC, JY, and CRV analyzed the data. AC, CRV, JY, and RVT wrote the manuscript. All authors contributed to the article and approved the submitted version.

## Conflict of Interest

The authors declare that the research was conducted in the absence of any commercial or financial relationships that could be construed as a potential conflict of interest.
