# Supplementary material for: Coral-Associated Viral Assemblages From the Central Red Sea Align With Host Species and Contribute to Holobiont Genetic Diversity
Source: Front Microbiol. 2020 Sep 30;11:572534. doi: 10.3389/fmicb.2020.572534 (PMC7561429; doi:10.3389/fmicb.2020.572534)
Supplement: Supplementary file 1 [file Data_Sheet_1.docx]

Supplementary File for:

**Coral-associated viral assemblages from the central Red Sea align with host species and contribute to holobiont genetic diversity**

Anny Cárdenas^1^, Jin Ye^2^, Maren Ziegler^3^, Jérôme P. Payet^4^, Ryan McMinds^5^, Rebecca Vega Thurber^6^, Christian R Voolstra^1,2^*

**Affiliations:**

^1^Department of Biology, University of Konstanz, 78457 Konstanz, Germany

^2^ Red Sea Research Center, Division of Biological and Environmental Science and Engineering (BESE), King Abdullah University of Science and Technology (KAUST), Thuwal, Makkah, Saudi Arabia

^3^ Justus Liebig University, Department of Animal Ecology & Systematics, Heinrich-Buff-Ring 26-32 IFZ, 35392 Giessen, Germany

^4^ Oregon State University, College of Earth, Ocean, and Atmospheric Sciences, 104 Admin Bldg, Corvallis OR, 97330, USA

^5^ Université Côte d’Azur, Center for Modeling, Simulation and Interactions, 1361 Route des Lucioles, 06560 Valbonne, France

^6^ Oregon State University, Department of Microbiology, 220 Nash Hall, Corvallis OR, 97330, USA

*Corresponding author: Christian R Voolstra, christian.voolstra@uni-konstanz.de

Department of Biology

University of Konstanz

D-78457 Konstanz, Germany

Phone/Fax: 0049-7531- 883860‬

**
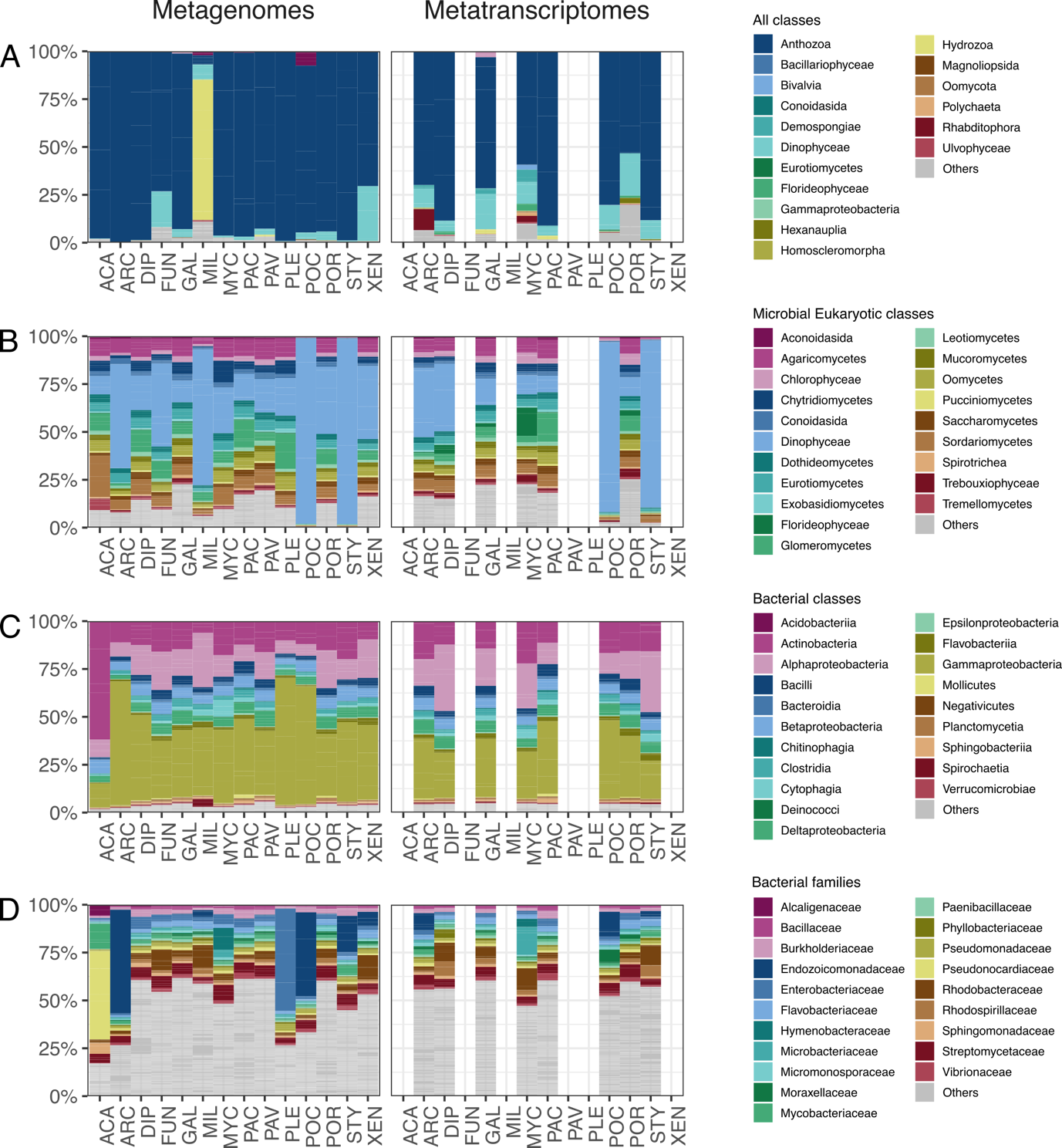
**

**Figure S1.** **Relative abundance of putative viral hosts.** **(A)** Overall taxonomic profiles of adaptor-trimmed paired-end reads obtained using CCMetagen. Relative abundances of quality-checked (cnidarian- and rRNA-free) paired-end reads classified to **(B)** microbial eukaryotic classes**,** **(C)** bacteria classes, and **(D)** bacterial families**.** Only the 20 most abundant taxa were considered in each plot. Coral species abbreviations: ACA - *Acanthastrea echinata*, ACR - *Acropora cytherea*, DIP- *Diploastrea heliopora*, FUN - *Fungia* sp., GAL - *Galaxea fascicularis*, MIL - *Millepora platyphylla*, MYC - *Mycedium elephantotus*, PAC - *Pachyseris speciosa*, PAV - *Pavona varians*, PLE - *Plerogyra sinuosa*, POC - *Pocillopora verrucosa*, POR - *Porites lutea*, STY - *Stylophora pistillata*, and XEN - *Xenia*

**
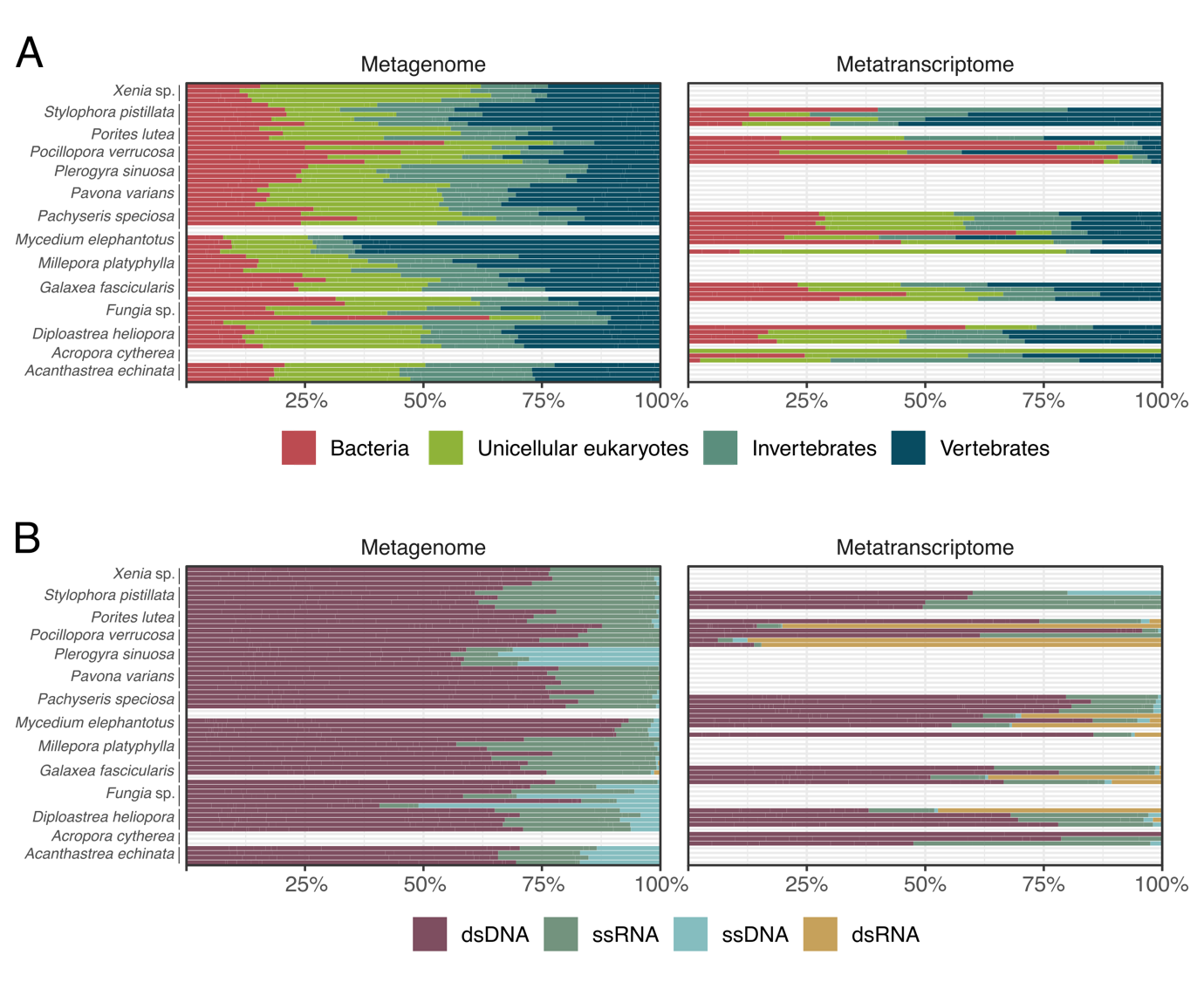
**

**Figure S2.** Relative abundance of viral types per **(A)** host and **(B)** nucleic acid**.** Only the 20 most abundant families are considered.


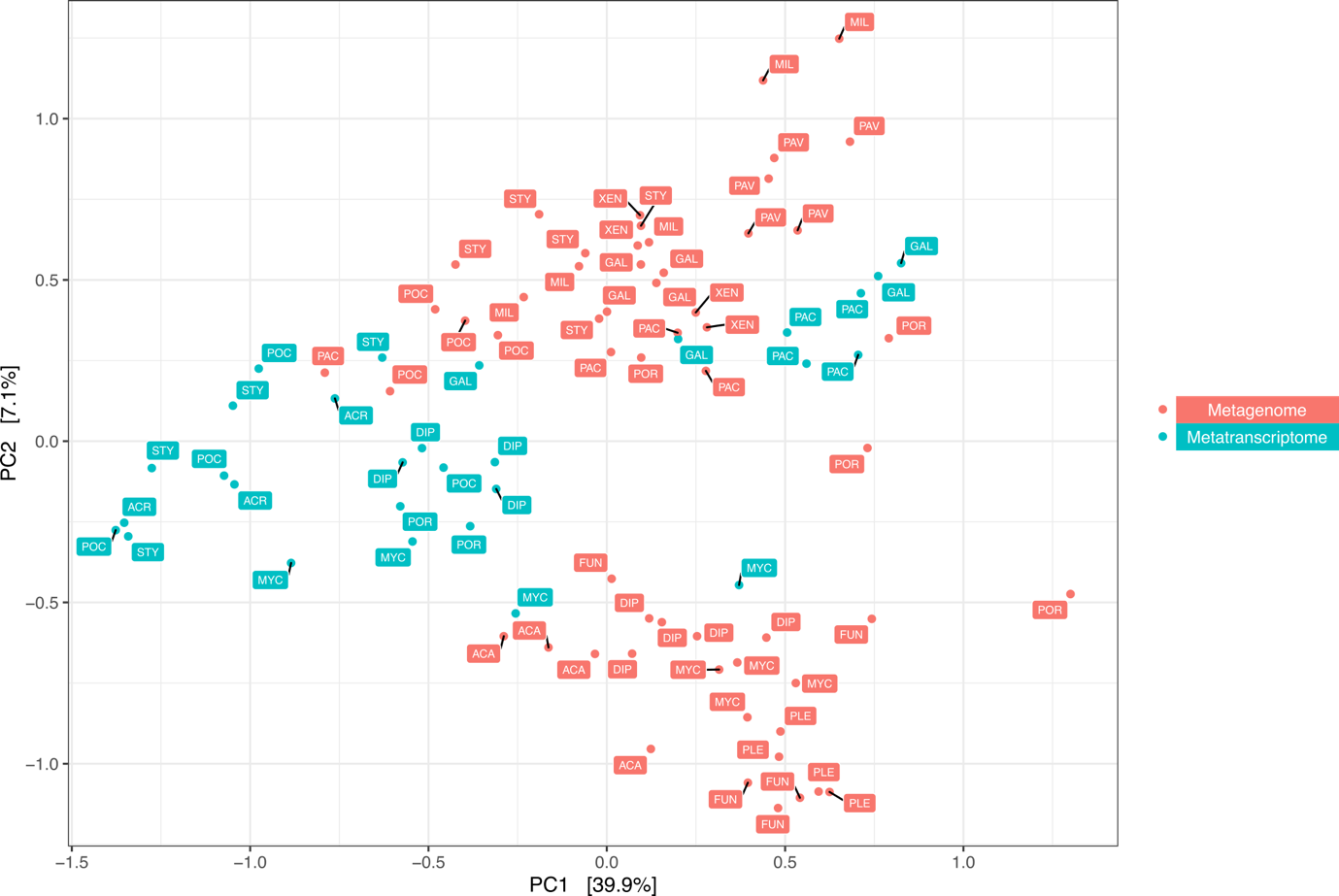


**Figure S3.** Differences in virome composition across metagenomes and metatranscritomes, Unconstrained redundancy analysis (RDA) represented in a Principal Component Analysis (PCA). Coral species abbreviations: ACA - *Acanthastrea echinata*, ACR - *Acropora cytherea*, DIP- *Diploastrea heliopora*, FUN - *Fungia* sp., GAL - *Galaxea fascicularis*, MIL - *Millepora platyphylla*, MYC - *Mycedium elephantotus*, PAC - *Pachyseris speciosa*, PAV - *Pavona varians*, PLE - *Plerogyra sinuosa*, POC - *Pocillopora verrucosa*, POR - *Porites lutea*, STY - *Stylophora pistillata*, and XEN - *Xenia*

**
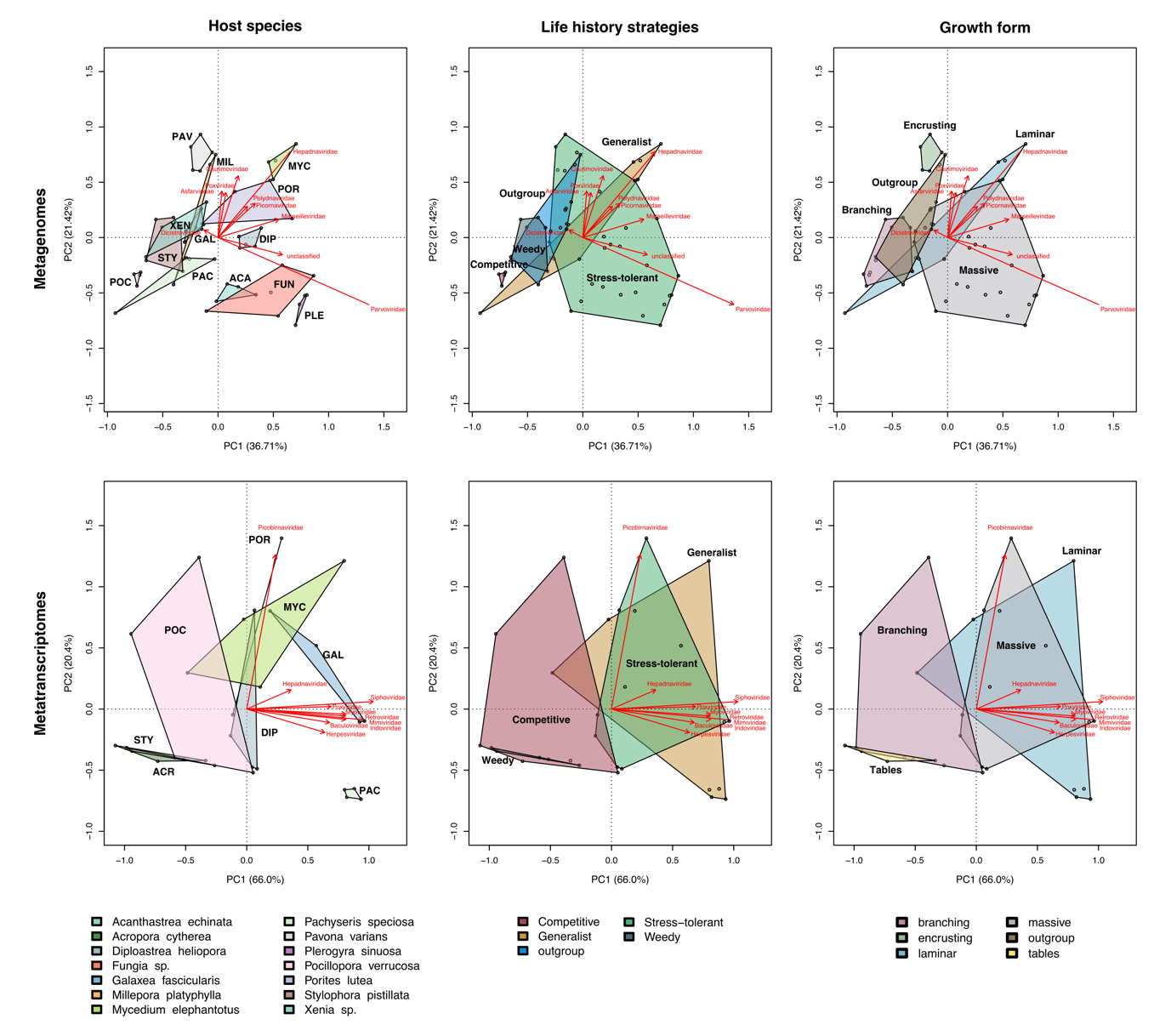
**

**Figure S4.** Relative contributions of viral families to differences in viromes across coral host species, growth forms and life history strategies. Scores for viral families were obtained by an unconstrained distance-based redundancy analysis (dbRDA) of Bray-Curtis dissimilarities and are indicated by biplot arrows in the Principal component analysis (PCA). Only the top 10 families with highest scores were used for the analysis. The percent variance explained by each axis is denoted in the axis label. Coral species abbreviations: ACA - *Acanthastrea echinata*, ACR - *Acropora cytherea*, DIP- *Diploastrea heliopora*, FUN - *Fungia* sp., GAL - *Galaxea fascicularis*, MIL - *Millepora platyphylla*, MYC - *Mycedium elephantotus*, PAC - *Pachyseris speciosa*, PAV - *Pavona varians*, PLE - *Plerogyra sinuosa*, POC - *Pocillopora verrucosa*, POR - *Porites lutea*, STY - *Stylophora pistillata*, and XEN - *Xenia*
